# FAM168A participates in the development of chronic myeloid leukemia via BCR-ABL1/AKT1/NFκB pathway

**DOI:** 10.1186/s12885-019-5898-4

**Published:** 2019-07-10

**Authors:** Xiaorong Liu, Huirong Mai, Hanfang Jiang, Zhihao Xing, Dong Peng, Yuan Kong, Chunqing Zhu, Yunsheng Chen

**Affiliations:** 1 Clinical laboratory, Shenzhen Children’s Hospital, No. 7019, Yitian Road, Shenzhen, Guangdong 518038 People’s Republic of China; 2 Division of Hematology and Oncology, Shenzhen Children’s Hospital, Shenzhen, Guangdong 518038 People’s Republic of China

**Keywords:** Chronic myeloid leukemia, FAM168A, Cell proliferation, AKT1, BCR-ABL1

## Abstract

**Background:**

Although the prognosis of chronic myeloid leukemia (CML) has dramatically improved, the pathogenesis of CML remains elusive. Studies have shown that sustained phosphorylation of AKT1 plays a crucial role in the proliferation of CML cells. Evidence indicates that in tongue cancer cells, FAM168A, also known as tongue cancer resistance-associated protein (TCRP1), can directly bind to AKT1 and regulate AKT1/NFκB signaling pathways. This study aimed to investigate the role of FAM168A in regulation of AKT1/NFκB signaling pathway and cell cycle in CML.

**Methods:**

FAM168A interference was performed, and the expression and phosphorylation of FAM168A downstream proteins were measured in K562 CML cell line. The possible roles of FAM168A in the proliferation of CML cells were investigated using in vitro cell culture, in vivo animal models and clinical specimens.

**Results:**

We found that the expression of FAM168A significantly increased in the peripheral blood mononuclear cells of CML patients, compared with normal healthy controls. FAM168A interference did not change AKT1 protein expression, but significantly decreased AKT1 phosphorylation, significantly increased IκB-α protein level, and significantly reduced nuclear NFκB protein level. Moreover, there was a significant increase of G2/M phase cells and Cyclin B1 level. Immunoprecipitation results showed that FAM168A interacts with breakpoint cluster region (BCR) -Abelson murine leukemia (ABL1) fusion protein and AKT1, respectively. Animal experiments confirmed that FAM168A interference prolonged the survival and reduced the tumor formation in mice inoculated with K562 cells. The results of clinical specimens showed that FAM168A expression and AKT1 phosphorylation were significantly elevated in CML patients.

**Conclusion:**

This study demonstrates that FAM168A may act as a linker protein that binds to BCR-ABL1 and AKT1, which further mediates the downstream signaling pathways in CML. Our findings demonstrate that FAM168A may be involved in the regulation of AKT1/NFκB signaling pathway and cell cycle in CML.

**Electronic supplementary material:**

The online version of this article (10.1186/s12885-019-5898-4) contains supplementary material, which is available to authorized users.

## Background

Chronic myelogenous leukemia (CML) is a malignant tumor formed by clonal proliferation of bone marrow hematopoietic stem cells. Due to the proliferation and infiltration of cloned leukemia cells in bone marrow and other hematopoietic tissues, normal hematopoietic function is inhibited. The clinical manifestations include anemia, hemorrhage, weight loss, upper abdominal discomfort, and splenomegaly. CML can occur in people of any age, with an annual global incidence of (1.6–2)/100,000 [1]. The incidence of CML increases with age. Although tyrosine kinase inhibitors have made a great breakthrough in the treatment of CML, little is known about the pathogenesis of CML.

More than 95% of CML patients have characteristic Philadelphia chromosome (Ph), which is produced by the translocation of chromosome 9 and 22 t (9;22)(q34;q11) [2, 3]. Studies have shown that the Philadelphia chromosome produces a fusion gene, which in turn encodes the BCR-ABL1 fusion protein. This enzyme has a sustained tyrosine kinase activity and it can promote phosphorylation of downstream signaling proteins such as AKT1, ERK, and STAT1. It can also increase the expression of oncogene c-Myc and apoptosis suppressor gene Bcl-2, and promote cell proliferation and survival [4–6]. However, it is still unclear how BCR-ABL1 protein promotes the sustained phosphorylation of AKT1 protein.

FAM168A, also known as tongue cancer resistance-associated protein 1 (TCRP1) was discovered in studies of multidrug resistance of tongue cancer cells. FAM168A gene is located on chromosome ll, and its protein consists of 235 amino acids with a molecular weight of 25 kDa [7]. Studies show that FAM168A mediates tumor cell cisplatin resistance and radiotherapy tolerance [7–11]. In one study researchers used human toxicology and drug resistance microarray (OHS-401) in combination with immunoprecipitation to detect the gene expression profiles in FAM168A-overexpression and FAM168A-knockout tongue cancer cell lines. They found that FAM168A directly binds to AKT1 [9]. Moreover, a decrease in AKT1 and NFκB expression was observed in FAM168A-deletion cells. These results indicate that FAM168A may promote tumor cell proliferation and reduce apoptosis via AKT1/NFκB signaling pathway [9, 11]. Here we investigated the impact of FAM168A interference on FAM168A expression and AKT1 phosphorylation in K562 CML cell line, in vivo animal model and clinical specimens. Our study provides theoretical and clinical basis for the development of new CML therapeutic targets.

## Methods

### Materials

RIPA lysis and extraction buffer was purchased from Beyotime Biotechnology Co., Ltd. (Jiangsu, China). PhosSTOP Phosphatase Inhibitor Cocktail Tablets were purchased from Roche (Shanghai, China). Nuclear protein extraction kit was purchased from Active Motif Co., Ltd. (Shanghai, China). Protease inhibitor cocktail set III was purchased from EMD Biosciences (Germany). FAM168A antibody [FAM168A (E-13)] was purchased from Santa Cruz (U.S.A.). AKT1 antibody, p-AKT1 antibody, β-actin antibody, HRP-conjugated secondary antibodies, goat anti-rabbit IgG and horse anti-mouse IgG were purchased from Cell Signaling (U.S.A.). Fetal bovine serum, IMEM media, and trypsin were purchased from Invitrogen (U.S.A.). BCA protein assay kit was purchased from Pierce Corporation (U.S.A.). SDS, Tris-Cl, EDTA, and acrylamide were purchased from Sigma (U.S.A.).

Human chronic myeloid leukemia cell line K562 was purchased from China Center for Type Culture Collection. K562 cells were cultured in IMEM media containing 10% fetal bovine serum at 37 °C with 5% CO_2_. Cells in logarithmic growth phase were selected.

Twenty-one male NOD/SCID mice, 5–6 weeks of age, weighing 25–30 g, were purchased from Nanjing University-Nanjing Institute of Biomedical Research (Qualification No.: 32002100001171), and were housed in specific-pathogen-free (SPF) laminar air flow rooms [equipment qualification certificate: SYXK (Guangdong) 2012–0119] at Shenzhen Institute of Advanced Technology. Animals had free access to food and water. The study was approved by the Laboratory Animal Ethics Committee of the Shenzhen Advanced Institute of the Chinese Academy of Sciences.

Three patients, 2 males (6–10 years old) and 1 female (6–10 years old), with CML hospitalized in the Department of Hematology and Oncology at our hospital from March 2018 to May 2018 were included. These patients were newly diagnosed with CML. The clinical features of patients are shown in the Additional file 1: Table S1. Peripheral blood (2 mL) were collected from these patients before treatment. During the same time period, 3 healthy children underwent annual physical examination were selected, including 2 males and 1 female, 6–9 years of age, and 2 mL of peripheral blood were collected. The study was approved by the hospital’s ethics committee. Informed consents were signed by the parents of every child before the blood collection.

### Methods

#### Specimens collection

Inclusion criteria for CML patients were as follows: patients who had typical elevated white blood cell counts and abnormalities in classification, splenomegaly with Ph chromosomes or their variant karyotypes, and BCR-ABL1 fusion gene positivity according to the clinical diagnosis. Inclusion criteria for healthy controls were as follows: healthy children who had no underlying diseases. 2 mL of peripheral blood was collected from the patients and the healthy controls using EDTAK2 anticoagulant tubes and stored at 4 °C until use.

#### Isolation of peripheral blood mononuclear cells

The whole blood was diluted with equal amount of D-Hank’s solution or PBS. 2 mL of the diluted blood was added along the wall of the tube into 1 mL of lymphocyte isolation solution and centrifuged at 400 x g for 20 min at 15 °C. The white lymphocyte layer was collected using a capillary pipette, placed in another centrifuge tube and washed with PBS for 3 times to collect the cells.

#### Cell culture

K562 cells were cultured in IMEM media (Thermo Fisher Scientific, U.S.A.) containing 10% fetal bovine serum (Thermo Fisher Scientific, U.S.A.), and incubated at 37 °C with 5% CO_2_. The cells in logarithmic growth phase were selected.

#### FAM168A siRNA interference

The siRNA sequence for the FAM168A exon (5′- GCCUCAUGUCAUCCACCAU − 3′) and the negative control siRNA sequence were designed and synthesized by Guangzhou Ruibo Biotechnology Co., Ltd. (Guandong, China). K562 cells were seeded into 6-well plates at a density of 3 × 10^5^ per well. Next day, the siRNAs and Lipofectamine 2000 were dissolved in serum-free media, mixed, and incubated at room temperature for 20 min. siRNAs/Lipofectamine 2000 mixtures were added to the cells, and cultured for 48 h.

#### RNA isolation and first strand cDNA synthesis

5 × 10^5^ PBMCs and K562 cells were collected and homogenized in 1 mL Trizol. 0.2 mL chloroform was added, vortexed, and centrifuged at 2,000 x g for 15 min at 4 °C. The upper aqueous phase was collected and 0.5 mL isopropanol was added. After high-speed centrifugation, the supernatant was discarded. The RNA pallet was washed with 75% ethanol and centrifuged to remove the supernatant. The pellet was dried and dissolved in DEPC-treated water. RNA concentration and quality were measured, and stored at − 70 °C. For reverse transcription, 2 U DNase I was added to 1 μg RNA and incubate at 37 °C for 30 min to remove the genomic DNA. EDTA was added to stop the reaction. Oligo (dT) primers and reverse transcriptase were added to the RNA and incubate at 42 °C for 60 min. The reaction was stopped by heating at 70 °C for 5 min.

#### Real-time quantitative PCR

A total of 20 μL of reaction system was used and 3 replicates were set for each sample. Real-time PCR program was as follows: 95 °C for 10 min; 95 °C for 15 s, 60 °C for 1 min, 40 cycles. The melting curve was plotted at 70 °C to 95 °C. The experiment was repeated three times. β-Actin was used as an internal reference control. The gene expression was calculated by comparative Ct method (2^-ΔΔCt^ method). The ΔΔCt was calculated as: ΔΔCt = [(target gene Ct - internal reference Ct) _treatment group_ - (target gene Ct - internal reference Ct) _control group_]. Real-time PCR detection primers for FAM168A were: 5′-GAACTCGTCTTC CTGTGGCA-3′ (FAM168A-F) and 5′-GGGGTGGAGCAGTGTTACTC-3′ (FAM168A-R). Detection primers for β-Actin were: 5′-CTCACCATGGATGATGATATCGC-3′ (β-Actin-F) and 5′-AGGAATCCTTCTGACCCATGC-3′ (β-Actin-R).

#### Nuclear protein extraction

K562 cells were cultured in 10-cm cell culture dishes. When the cells reached 80% confluence, the media were removed, and the cells were washed with 5 mL of ice-cold PBS containing phosphatase inhibitor. After centrifugation, the supernatant was removed. Nuclear protein extraction kit was used to extract nuclear protein. In brief, the cells were gently resuspended in 500 μL 1× hypotonic buffer, transferred to a 1.5 mL ice-cold centrifuge tube, and incubated on ice for 15 min. 25 μL detergent solution was added, vortexed for 10 s, and centrifuged at 14,000 x g for 30 s at 4 °C. The pellet was resuspended in 50 μL complete lysis buffer, vortexed for 10 s, incubated on ice for 30 min, vortexed for 30 s, and centrifuged at 14,000 x g for 10 min at 4 °C. The supernatant was used for Western blot analysis.

#### Western blot

6 × SDS loading buffer [0.35 M Tris-HCl, pH 6.8, 36% glycerol, 0.012% bromophenol blue, 10.28% SDS (w/v), 0.6 M DTT] was added to10 μg protein, boiled at 100 °C for 5 min, and cooled on ice. The proteins were loaded onto a 10% SDS-PAGE, and separated by electrophoresis. The proteins were transferred to PVDF membranes. The membranes were blocked with 5% skim milk in TBST overnight at 4 °C, and incubated with primary antibodies for 2 h. The membranes were washed three times (5 min each) with TBST, and then incubated with the corresponding secondary antibodies for 1 h. The membranes were washed three times with TBST, and then rinsed three times with ddH_2_O. The membranes were reacted with ECL chemiluminescence detection reagents in dark, and the X-ray films were developed and imaged.

#### Bioinformatics analysis of FAM168A gene motif

The protein sequence of human FAM168A was downloaded from the GenBank (number: NP_055974.1) from the National Center for Biotechnology Information (NCBI) database, and the motif analysis of FAM168A was performed using Scansite database (http://scansite.mit.edu/) [12].

#### Immunoprecipitation

K562 cells were cultured in a 10 cm dish. After the cells reached 100% confluence, the media were removed and the cells were washed once with PBS. 400–500 μL of RIPA lysis buffer (50 mM Tris-Cl, pH 7.4; 150 mM NaCl; 1% Triton X-100; 1% sodium deoxycholate; 0.1% SDS; 1 mM sodium orthovanadate; 10 mM sodium fluoride; 1% protease inhibitor cocktail) was added to the cells and incubated on ice for 10 min. After sonication, the lysates were centrifuged at 13000 x g for 10 min at 4 °C, and the supernatants were collected. A small amount of supernatants were used as an “Input sample” for Western blot analysis. The remaining supernatants were divided into four equal portions. The first three portions were incubated with 1–2 μg of FAM168A, c-ABL1, or AKT1 antibody overnight at 4 °C, respectively. The fourth portion was incubated with the same amount of rabbit IgG as a negative control. The next day, 20 μL of protein G beads were added to each tube and incubated at room temperature for 30 min. The magnetic beads were washed three times with RIPA lysis buffer. 40 μL of 2 × SDS loading buffer was added to the beads, heated at 95–100 °C for 5 min, and centrifuged at 13000 x g for 1 min to collect the supernatant. The supernatant and the Input sample were analyzed by Western blot.

#### Flow cytometry

K562 cells were seeded into 6-well plates, transfected with FAM168A siRNA or control siRNA, and cultured for 48 h. Cells were collected and washed with PBS. Cells were resuspended in 70% ice-cold ethanol, fixed for 2 h, and centrifuged to remove supernatants. The cells were washed twice with 0.5% BSA and 0.1% Triton X-100 in PBS. Propidium iodide was added and incubated for 30 min, and the cell cycle DNA content was measured by flow cytometry (Beckman Coulter).

#### CML mouse model

Twenty-one male NOD/SCID mice, 5–6 weeks old, weighing 25–30 g, were housed in a SPF laminar flow chamber. The mice were randomized into 3 groups of 7: K562/Control group, K562/siFAM168A group and negative control group. The mice in K562/siFAM168A and K562/Control groups were injected with K562 cells transfected with FAM168A siRNA or control siRNA, respectively, via the tail vein. The cell concentration was 5 × 10^6^ / mouse, and the injection volume was 0.2 mL. The mice in the negative control group received saline injection (0.2 mL/mouse) via the tail vein. The mice in negative control group were sacrificed by neck dislocation at the end of experiment.

#### Survival analysis

The color of the fur, body weight, tumor growth, and survival duration of NOD/SCID mice in each group were recorded, and the tumor formation rate and survival rate were compared.

#### Statistical analysis

The experimental data were analyzed by SPSS19.0 statistical software, and the data were expressed as mean ± SD (standard deviation). Student t-test was used for two-group comparison and *p* < 0.05 was considered statistically significant.

## Results

### FAM168A expression in PBMCs and K562 cells

**Fig. 1:**
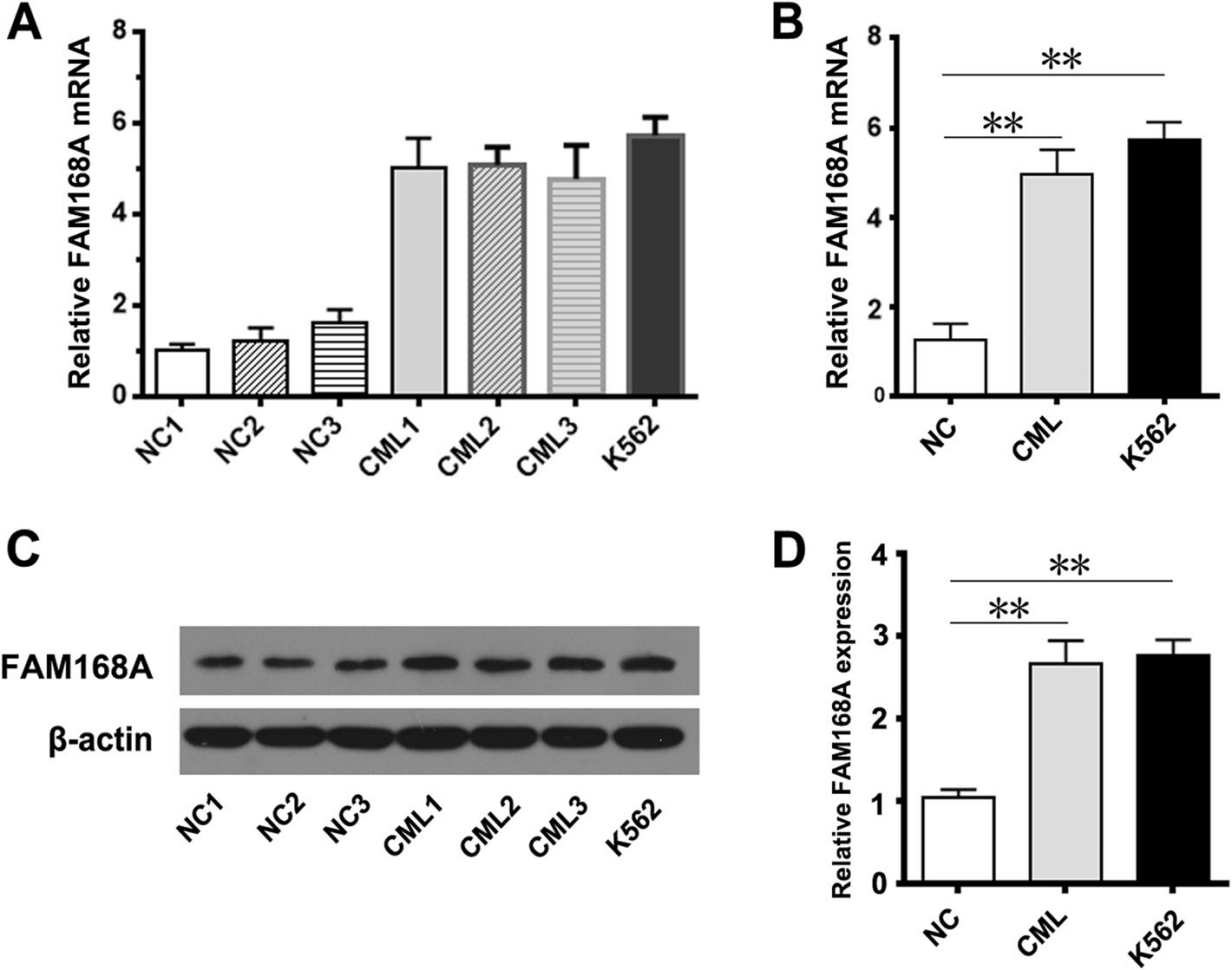
FAM168A expression in PBMCs and K562 cells. **a** PBMCs were isolated from the peripheral blood of three healthy children and three CML patients. The FAM168A mRNA expression was higher in CML patients and K562 cells. **b** The mean FAM168A mRNA expression was significantly higher in CML patients and K562 cells competed to healthy controls. **c** Western blot images of FAM168A protein expression in PBMCs from CML patients, healthy controls and K562 cells. **d** The mean FAM168A protein expression in CML patients, healthy controls and K562 cells. Data were presented as mean ± SD from three independent experiments. **p < 0.01

FAM168A expression in PBMCs of CML patients and healthy controls, as well K562 cells were detected by real-time quantitative PCR and Western blot. We found that the FAM168A mRNA and protein expressions in PBMCs of CML patients, as well as K562 cells, were significantly higher compared to healthy controls, suggesting that FAM168A may be involved in the CML development (Fig. 1).

### FAM168A is involved in the regulation of AKT1/NFκB signaling pathway in K562 cells

**Fig. 2:**
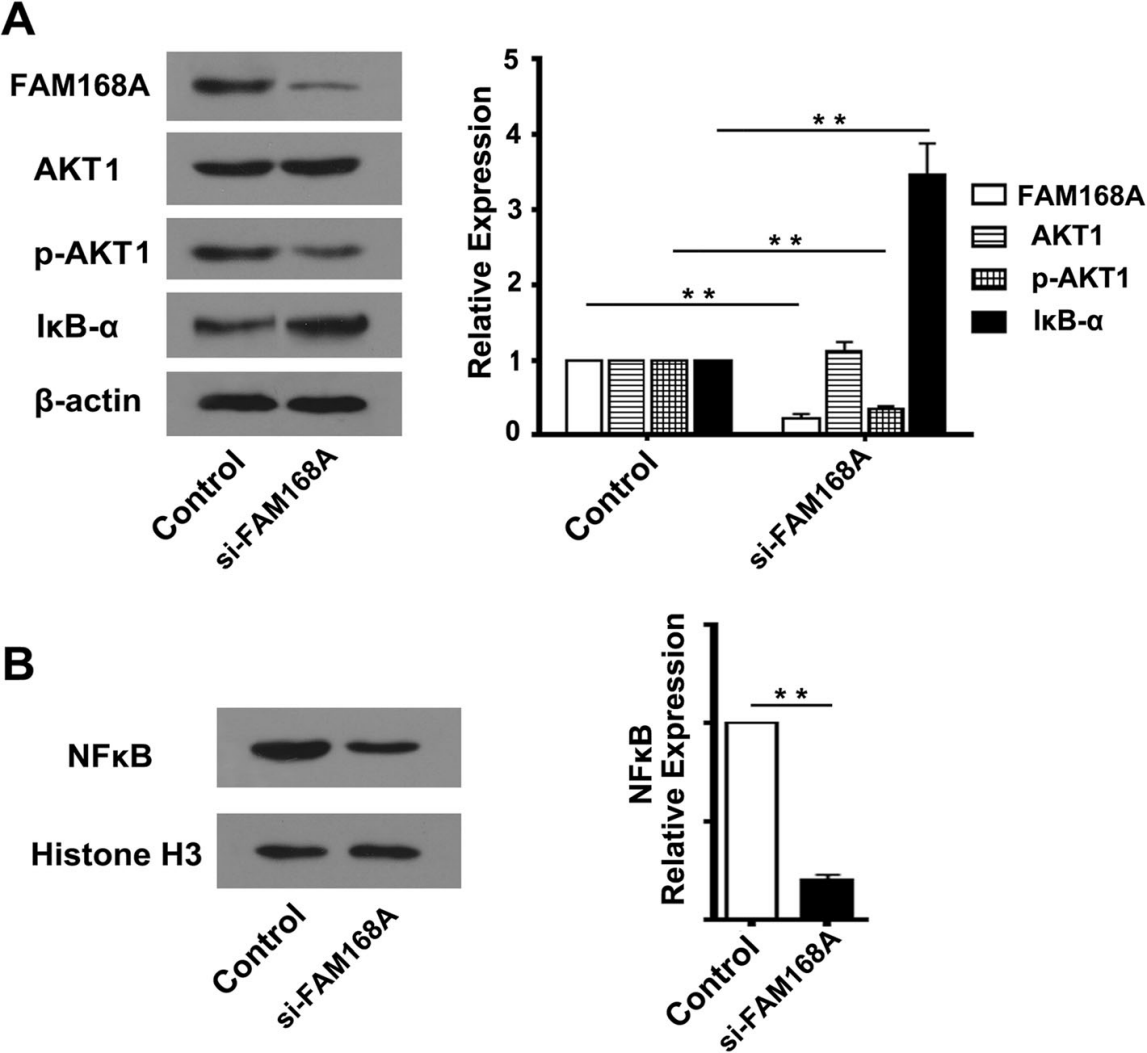
FAM168A may be involved in the regulation of AKT1/NFκB signaling pathway in K562 cells. K562 cells were transfected with FAM168A siRNA or negative control siRNA, respectively. **a** The changes in AKT1, p-AKT1 and IκB-α levels in whole cell lysates. **b** The change in NFκB protein in nuclear extracts. Data were presented as mean ± SD from three independent experiments. **p < 0.01

We investigated the impact of FAM168A interference on the expression of AKT1, phosphorylated-AKT1 (p-AKT1) and IκB-α. We found that FAM168A interference did not change AKT1 protein level in K562 cells, but p-AKT1 level was significantly decreased, and IκB-α protein was significantly increased (Fig. 2a).

To further explore the role of FAM168A in NFκB signaling pathway, K562 cells were treated with FAM168A siRNA or negative control siRNA. The nuclear protein was extracted for Western blot analysis. The results showed that FAM168A interference significantly decreased the nuclear NFκB level (Fig. 2b), suggesting that FAM168A may be involved in the regulation of AKT1/NFκB signaling pathway in K562 cells.

### Bioinformatics analysis of FAM168A functional motifs

**Fig. 3:**
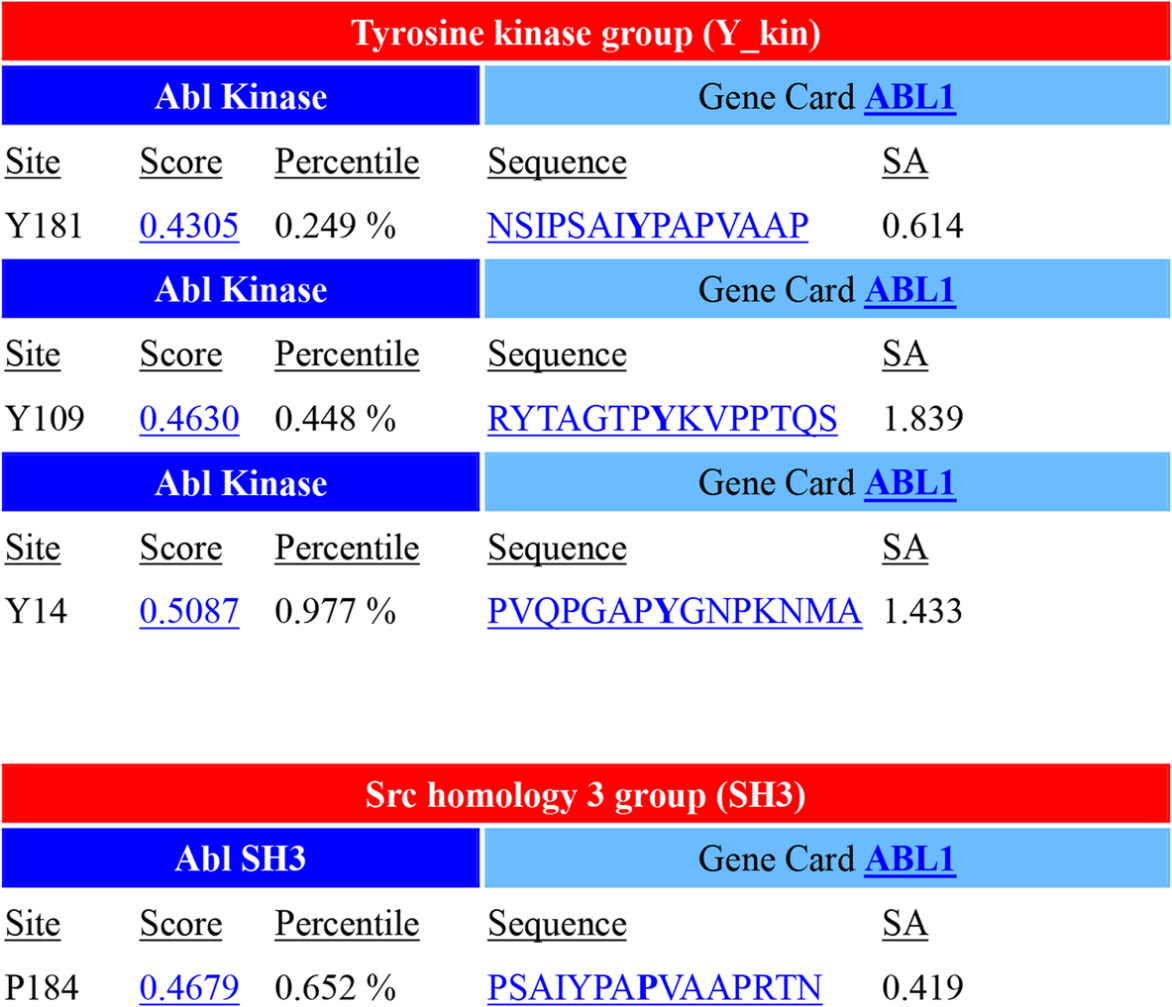
FAM168A functional motif prediction using the Scansite database (http://scansite.mit.edu/)

The functional motif of FAM168A was analyzed using online database Scansite. Score and percentile were used as two indicators. The higher the score, the greater the deviation from the best match. Percentile indicates the ranking of a predictive sequence score for that known motif. The predicted results of the functional motifs of FAM168A are shown in Fig. 3. We found that there are multiple ABL1 kinase motifs and ABL1 SH3 motifs in the FAM168A protein sequence, suggesting that FAM168A may interact with ABL1 kinase.

### FAM168A interacts with BCR-ABL1 and AKT1

**Fig. 4:**
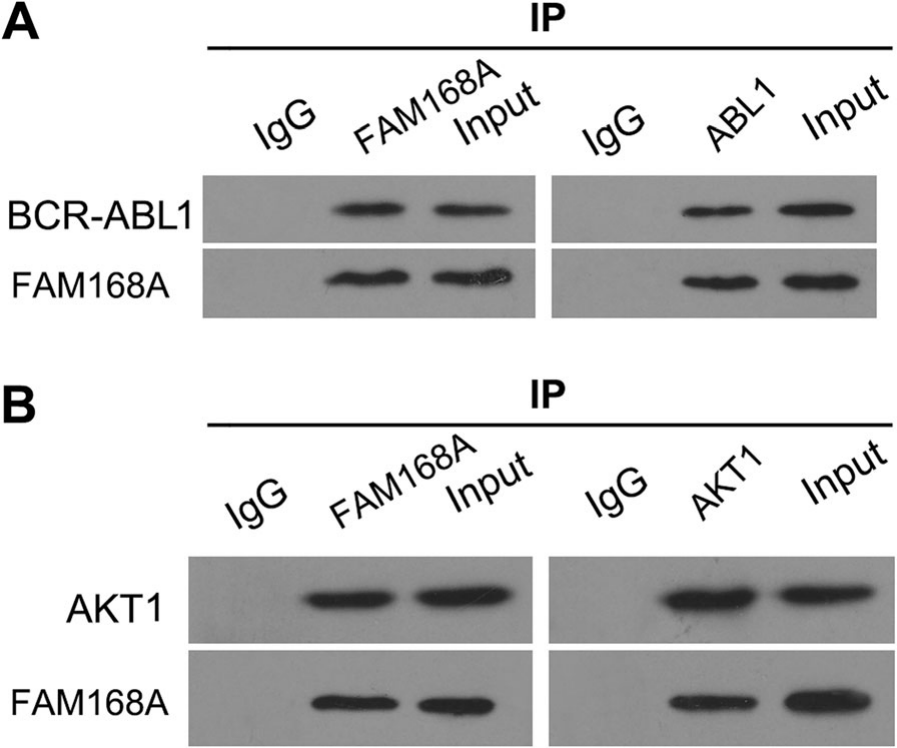
FAM168A interacts with BCR-ABL1 and AKT1. K562 cells were seeded in a 10 cm dish. Cells were harvested and lysed with RIPA buffer. **a** The lysates were incubated with normal mouse IgG-conjugated agarose (Ig G), anti-FAM168A antibody-conjugated agarose (FAM168A) or anti-ABL1 antibody-conjugated agarose (ABL1). The immunoprecipitants and cell lysates (Input) were electrophoresed and immunoblotted with ABL1 and FAM168A. **b** The K562 lysates were incubated with normal mouse IgG-conjugated agarose (Ig G), anti-FAM168A antibody-conjugated agarose (FAM168A) or anti-AKT1 antibody-conjugated agarose (AKT1). The immunoprecipitants and cell lysates (Input) were electrophoresed and immunoblotted with AKT1 and FAM168A

The interaction of FAM168A and AKT1 or BCR-ABL1 was detected using immunoprecipitation. The results show that BCR-ABL1 protein and AKT1 protein were detected in the immunoprecipitate using FAM168A antibody. FAM168A protein was also detected in the immunoprecipitate using c-ABL1 and AKT1 antibodies, confirming that FAM168A interacts with BCR-ABL1 and AKT1 (Fig. 4).

### FAM168A interference affects K562 cell cycle

**Fig. 5:**
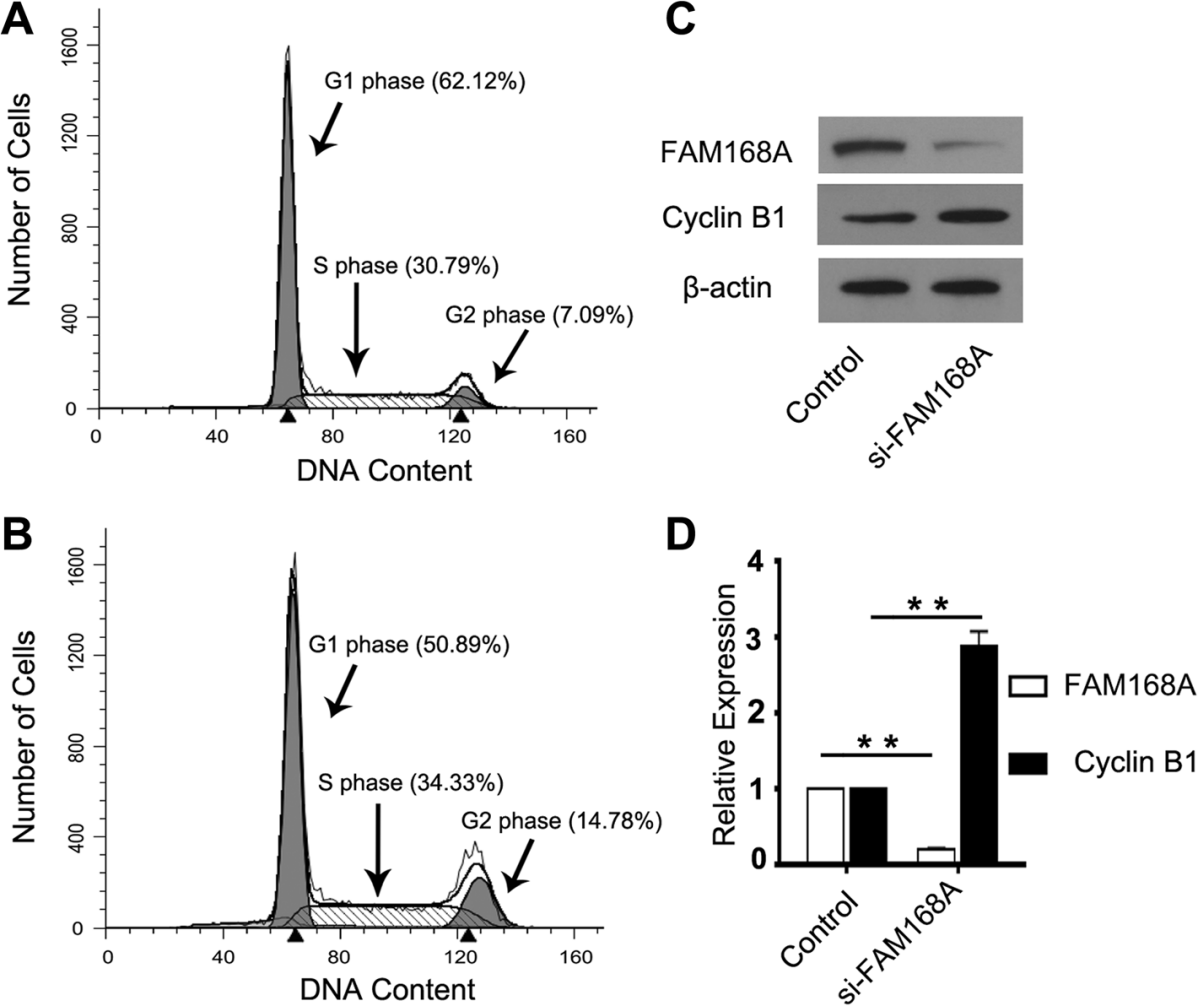
FAM168A interference affects K562 cell cycle. The K562 cells were transfected with FAM168A siRNA or negative control siRNA, respectively, and cultured for 48 h. Cells were harvested for flow cytometry and Western blot analysis. **a** Flow cytometric results of K562 cells transfected with the negative control siRNA for 48 h. **b** Flow cytometric results of K562 cells transfected with FAM168A siRNA for 48 h. **c** Western blot images of FAM168A and Cyclin B1 in K562 cells transfected with FAM168A siRNA or negative control siRNA. **d** Quantitative results of protein levels of FAM168A and Cyclin B1 in K562 cells transfected with FAM168A siRNA or negative control siRNA. Data were presented as mean ± SD from three independent experiments. **p < 0.01

To investigate whether FAM168A influences the cell cycle in K562 cells, K562 cells were transfected with FAM168A siRNA or negative control siRNA, respectively. Flow cytometry was used for cell cycle detection. The results show that the proportion of G2/M phase cells increased from 7.09 to 14.78%, suggesting that FAM168A interference induced G2/M arrest (Fig. 5a, b). Western blot analysis further confirmed that the expression of Cyclin B1, an important cyclin in the G2/M phase, increased after FAM168A interference (Fig. 5c).

### FAM168A interference prolongs the survival duration and reduces the tumor formation in K562 cell mouse model

**Fig. 6:**
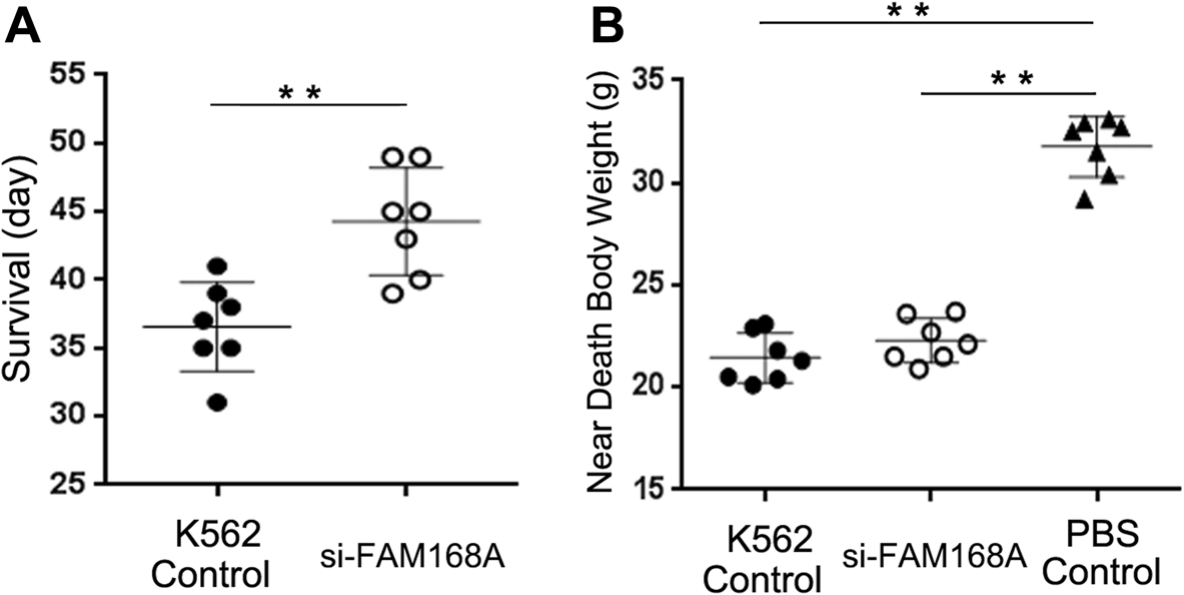
Survival analysis. **a** The survival duration is significantly higher in the K562/si-FAM168A mice compared to K562/Control mice. **b** The near death body weight in the K562/Control and K562/si-FAM168A mice was significantly lowers compared to the PBS Control mice. Error bars indicate the mean ± SD. **p < 0.01

**Fig. 7:**
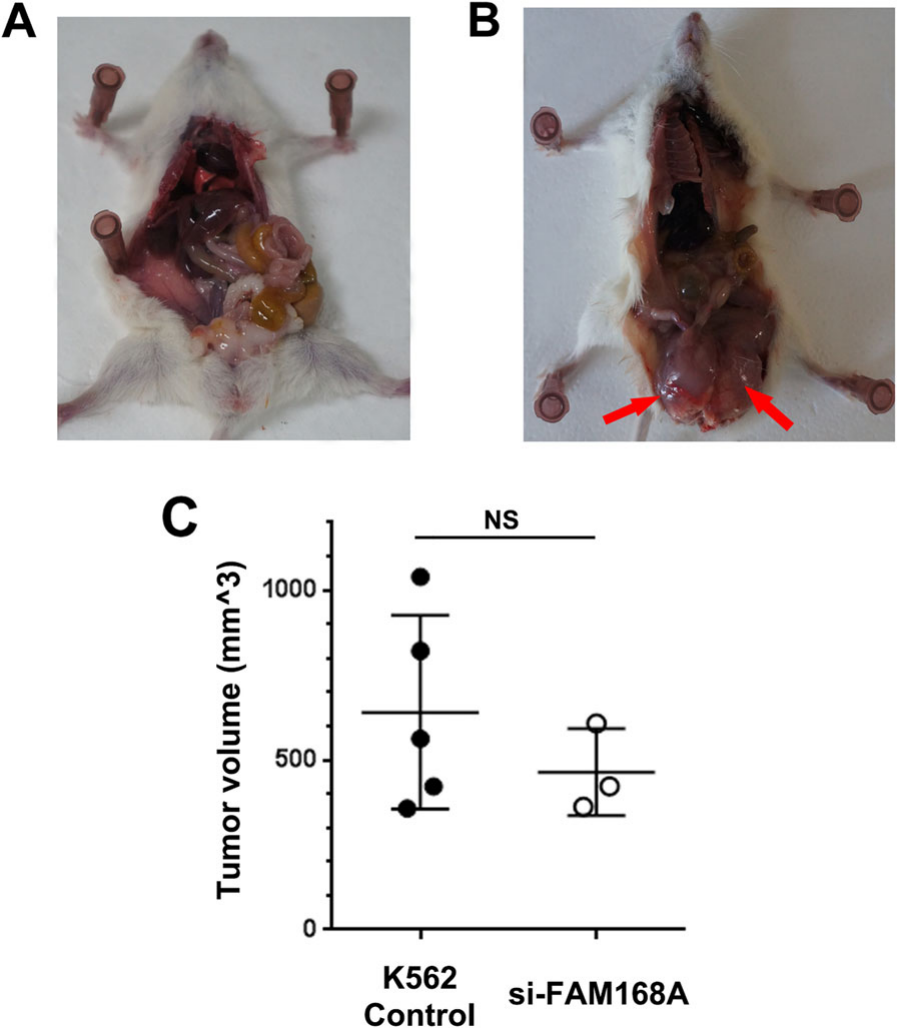
Tumor formation in the K562/si-FAM168A mice and K562/Control mice. **a** Schematic representation of a mouse in the PBS Control group. There was no tumor mass in the PBS Control mice. **b** Schematic representation of a mouse in the K562/Control group. The tumors are indicated by the red arrows. **c** The tumor volume in K562/si-FAM168A group and K562/Control group. NS: non-significant

The survival duration of the 7 mice in the K562/Control group was 31, 35, 35, 37, 38, 39, and 41 days, respectively. The survival duration for the 7 mice in the K562/siFAM168A group were 39, 40, 43, 45, 45, respectively. The mean survival duration was significantly longer in the K562/siFAM168A group (*p* < 0.01) (Fig. 6a). The mice in the PBS control group were active and had smooth fur; while the mice in the K562/Control and K562/siFAM168A groups were emaciated, hypoactive, and had dull fur. The near-death body weights of the K562/Control and K562/siFAM168A groups were significantly lower, compared to the negative control group (*p* < 0.01) (Fig. 6b). In the K562/Control group and the K562/siFAM168A group, there were 5 and 3 mice that had macroscopic tumors, and the tumor formation rates were 71.4 and 42.8%, respectively (Fig. 7). The PBS Control group had no tumor-bearing mouse. The above results indicate that the FAM168A interference can prolong the survival duration of mice in K562 cell model and reduce the tumorigenesis rate.

### Clinical specimens verify the role of FAM168A in CML

**Fig. 8:**
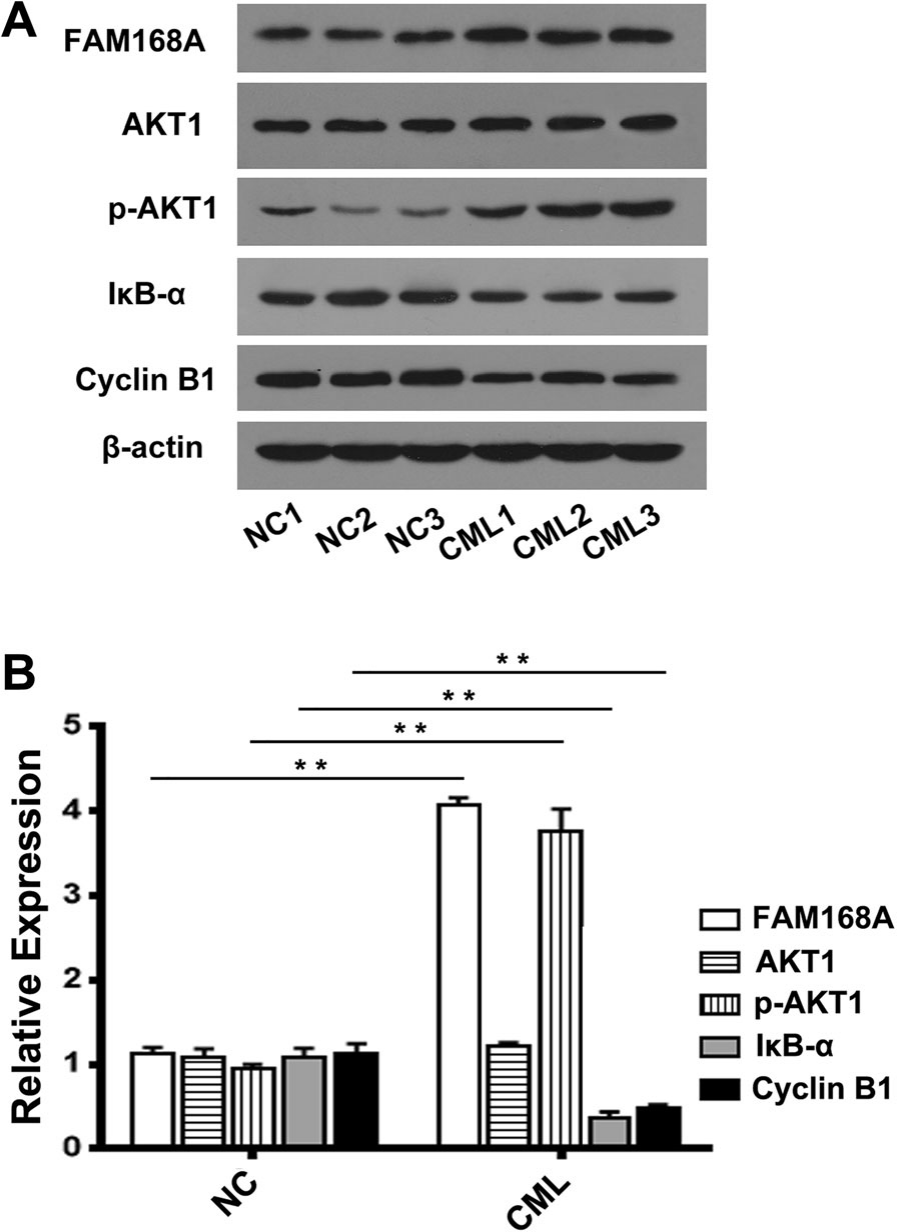
Clinical specimens verify the role of FAM168A in CML. PBMCs were isolated from the peripheral blood of 3 healthy controls (NC1, NC2, NC3) and 3 CML patients (CML1, CML2, and CML3). Proteins were extracted and FAM168A, AKT1, p-AKT1, IκB-α, and Cyclin B1were detected by Western blot. **a** The Western blot images of FAM168A, AKT1, p-AKT1, IκB-α and Cyclin B1. **b** the average expression of FAM168A, AKT1, p-AKT1, IκB-α and Cyclin B1. Data were presented as mean ± SD from three independent experiments. **p < 0.01

PBMCs were isolated from peripheral blood of 3 healthy controls and 3 CML patients. The levels of FAM168A, AKT1, p-AKT1, IκB-α and Cyclin B1 were measured using Western blot. The results show that the expression of FAM168A was elevated in PBMCs of CML patients. There was no difference in the expression of AKT1 protein, but the p-AKT1 protein was significantly increased in CML patients. Moreover, the IκB-α and Cyclin B1 (an important cyclin in the G2/M phase) were significantly decreased in CML patients (Fig. 8). These results are consistent with the cell study, suggesting that FAM168A may be involved in the regulation of AKT1/NFκB signaling pathway and cell cycle in CML.

## Discussion

In this study, we found that FAM168A mRNA and protein expressions were significantly increased in PBMCs of CML patients and in K562 cells. FAM168A interference did not change AKT1, but significantly decreased p-AKT1, significantly increased IκB-α, and significantly decreased nuclear NFκB. There is a reduction in G2/M phase cells and a significant increase of Cyclin B1, an important cyclin in the G2/M phase. We also found that there are multiple ABL1 kinase motifs and ABL1 SH3 motifs in the FAM168A protein sequence. Immunoprecipitation results confirmed that FAM168A interacts with BCR-ABL1 and AKT1, respectively. Animal experiments showed that FAM168A interference can prolong the survival duration and reduce the tumorigenesis rate in K562/siFAM168A mice, suggesting that FAM168A may promote the growth and metastasis of CML cells. The results of clinical specimens showed that the expression of FAM168A significantly increased in PBMCs of CML patients. Although there was no difference in AKT1, p-AKT1 significantly increased, while IκB-α and Cyclin B1 significantly decreased.

CML is a hematologic malignancy with abnormal hematopoietic stem cells. Although a large number of studies have been carried out, the mechanism of CML remains unclear [4]. Guo Xiao et al. [13] found that the expression of ERK, p38, p27, Cyclin E, and Cyclin D2 in CML cells increased, which accelerated cell cycle progression and cell proliferation, leading to the occurrence of CML. Li et al. [14] found that Bcl-2 and cyclin E1 mediate the proliferation and carcinogenesis of CML. FAM168A is a gene discovered in tumor drug resistance study. Studies have shown that FAM168A may mediate tumor cell proliferation and reduce apoptosis through AKT1/NFκB signaling pathway [8, 11]. In order to study whether FAM168A participates in the development of CML, we first examined the expression of FAM168A in CML cells and found that FAM168A mRNA and protein expressions increased in PBMCs of CML patients. FAM168A interference inhibited K562 cell proliferation and significantly reduced AKT1 phosphorylation, suggesting that FAM168A may mediate CML cell proliferation through AKT1 phosphorylation.

The dysregulation of AKT1/NFκB signaling pathway has a significant impact on the development of multiple tumors [15]. Over-activation of AKT1 can induce the mutation of the catalytic subunit of PI3K, leading to the occurrence and progression of tumors [16]. The AKT1/NFκB signaling pathway in CML cells is abnormally activated, which inhibits apoptosis and promotes malignant proliferation of CML cells [17]. Li et al. [18] found that abnormal activation of the PI3K/AKT1/NF-κB signaling pathway suppressed the activation and proliferation of T lymphocytes, leading to impaired immune regulation. Zhang et al. [19] found that the AKT1/NFκB signaling pathway mediates drug resistance by phosphorylating glycoproteins in CML cells. Our study found that there was no difference in the expression of AKT1 in K562 cells after FAM168A interference; however, AKT1 phosphorylation, IκB degradation and nuclear NFκB significantly decreased. Moreover, FAM168A expression in CML patients was higher compared to normal controls. There was no significant difference in AKT1 expression, however, AKT1 phosphorylation and IκB degradation increased. These results indicate that FAM168A may affect the proliferation of CML cells through the AKT1/NFκB signaling pathway.

**Fig. 9:**
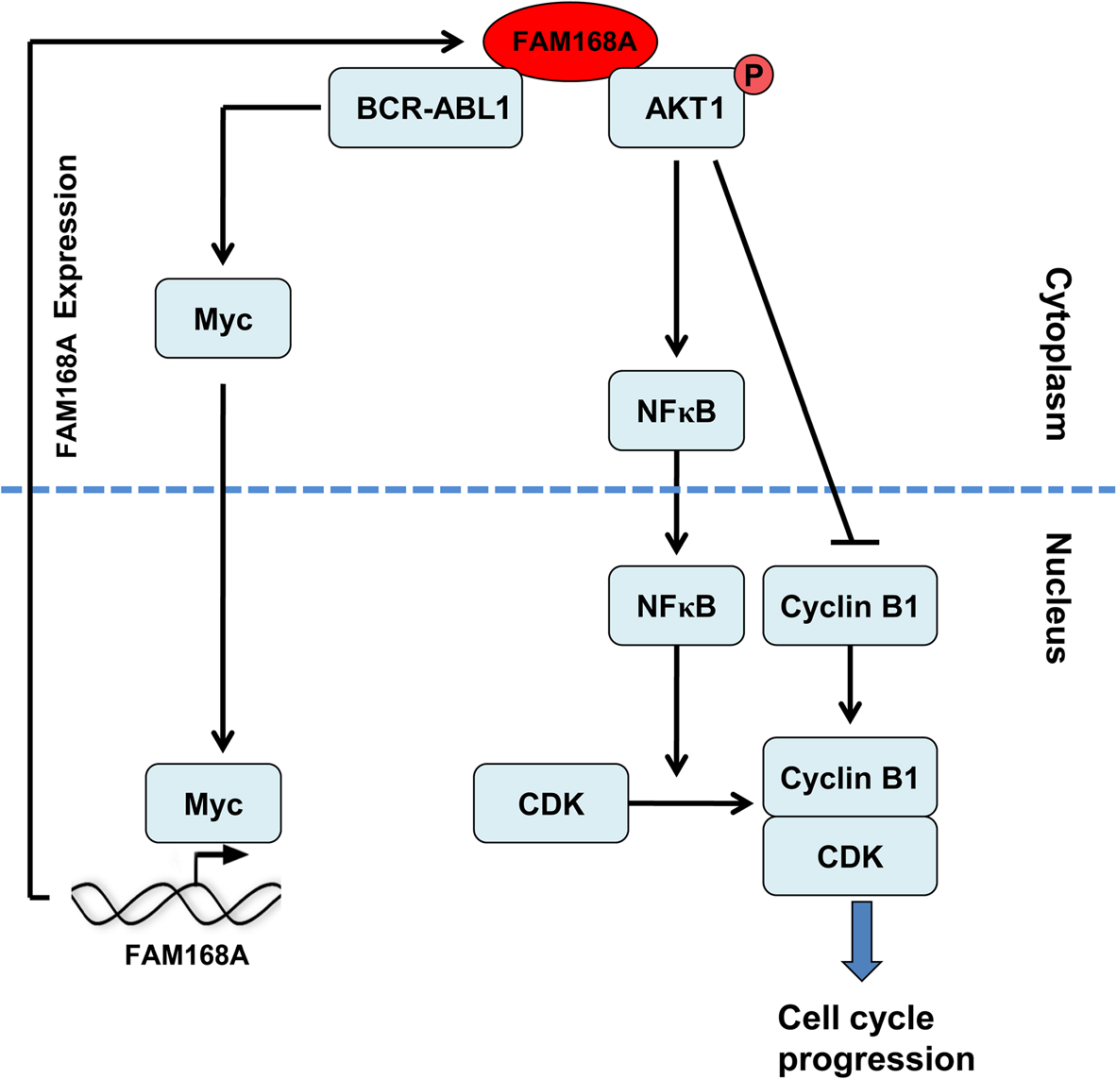
FAM168A participates in the development of CML. FAM168A may act as a linker protein to bind to BCR-ABL1 and AKT1, which mediates the transmission of AKT1/NFκB signal

The BCR-ABL1 fusion protein in CML patients can promote the sustained phosphorylation of a series of downstream signaling proteins such as AKT1, ERK and STAT1, as well as increase the expression of oncogene c-Myc and apoptosis suppressor gene Bcl-2. Cell proliferation and survival play a central role in the malignant proliferation of CML cells [4–6]. We found that FAM168A may be involved in the regulation of AKT1 phosphorylation in K562 cells. Bioinformatic analysis showed that there are multiple ABL1 kinase motifs and ABL1 SH3 motifs in the sequence of FAM168A protein. Immunoprecipitation results confirmed that FAM168A can bind to BCR-ABL1 and AKT1protein, respectively. We speculate that FAM168A may act as a linker protein that binds to both BCR-ABL1 and AKT1, and thus participate in BCR-ABL1 protein-mediated sustained phosphorylation of AKT1 protein in CML (Fig. 9). BCR-ABL1 fusion protein can increase the expression of c-Myc [20]. Recent studies have shown that c-Myc can be used as a transcription factor to further increase FAM168A expression [21]. Therefore, BCR-ABL1 fusion protein may regulate FAM168A expression via a positive feedback mechanism. FAM168A acts as a linker protein and participates in the BCR-ABL1/AKT1/NFκB signaling pathway, which further affects cell apoptosis and cell cycle. However, the underlying mechanisms need to be further investigated. Studies have shown that BCR-ABL1 protein expression is also found in patients with acute myeloid leukemia and acute lymphoblastic leukemia [20]. 2–5% of CML patients do not have typical Philadelphia chromosomes [3]. In our future study we will investigate whether FAM168A also plays a role in these diseases.

### The limitation of this study

The size of clinical samples is small in this study. In our future study, we will examine the relationship between FAM168A and BCR-ABL1/AKT1/NFκB signaling pathways using more clinical samples.

## Conclusion

This study revealed that FAM168A may act as a linker protein that binds to BCR-ABL1 and AKT1. The activation of AKT1/NFκB signaling pathway will promote the proliferation of CML cells. The discovery of our study provides a new target for the diagnosis and treatment of CML.

## Additional file

Additional file 1: **Table S1.** Clinical features of patients with CML. (DOC 29 kb)

## Data Availability

The datasets used and/or analyzed during the current study are available from the corresponding author on reasonable request.

## Abbreviations

CML: Chronic myeloid leukemia
FAM168A: Tongue cancer resistance-associated protein
NCBI: National Center for Biotechnology Information
Ph: Philadelphia chromosome
SPF: Specific-pathogen-free
