# Supplementary material for: FAM168A participates in the development of chronic myeloid leukemia via BCR-ABL1/AKT1/NFκB pathway
Source: BMC Cancer. 2019 Jul 10;19:679. doi: 10.1186/s12885-019-5898-4 (PMC6617578; doi:10.1186/s12885-019-5898-4)
Supplement: Supplementary file 1 — Table S1. Clinical features of patients with CML. (DOC 29 kb) [file 12885_2019_5898_MOESM1_ESM.doc]

Additional file 1: Table S1 Clinical features of patients with CML

| Patient | Age  (Years) | Disease entity | Chromosome abnormalities |
| --- | --- | --- | --- |
| 1 | 6-10 | Chronic phase | 46, XX, t(9:22) (q34:q11) [10] |
| 2 | 6-10 | Chronic phase | 46, XY, t(9:22) (q34:q11) [7] |
| 3 | 6-10 | Chronic phase | 46, XY, t(9:22) (q34:q11) [4] |
